# *LuciA-15* – a real-world prospective study of PARP inhibitors for the treatment of patients with HER-2 negative metastatic breast cancer with germline and/or somatic mutation of BRCA genes or homologous recombination repair related genes

**DOI:** 10.3332/ecancer.2023.1634

**Published:** 2023-11-21

**Authors:** Fernando E Petracci, Cynthia Villarreal-Garza, Facundo Argañaraz, Gonzalo Gómez Abuin, José Peñaloza, Marcos Ariel Flores, Luciano Piazzoni, Cecilia Riggi, Lucía Fabiano, Lucía González, Belén Cieplinski, Sergio Rivero, Ernesto Korbenfeld, Pablo Mandó

**Affiliations:** 1 Instituto Alexander Fleming, 1426, Buenos Aires, Argentina; 2 Hospital Zambrano Hellion, 64550, Monterrey, México; 3 Instituto Médico de Alta Complejidad, 4400, Salta, Argentina; 4 Hospital Alemán, 1118, Buenos Aires, Argentina; 5 Centro Oncológico Integral, 2800 Neuquén, Argentina; 6 Hospital Privado de Comunidad, 7600, Mar del Plata, Argentina; 7 Hospital Italiano, 1181, Buenos Aires, Argentina; 8 Hospital Municipal de Chivilcoy, Centro Santa María, 6620, Chivilcoy, Argentina; 9 COIR Fundación Centro Oncológico de Integración Regional, 5500, Mendoza, Argentina; 10 Centro Medico Accord Monserrat, 1086, Buenos Aires, Argentina; 11 Hospital Británico, 1280, Buenos Aires, Argentina; 12 CEMIC, Centro de Educación Médica e Investigaciones Clínicas, 1431, Buenos Aires, Argentina

## Abstract

**Background:**

Poly(adenosine diphosphate-ribose) polymerase inhibitors (PARPi) improve progression free survival among patients with HER2 negative (HER2-ve) advanced breast cancer (ABC) and a BRCA1 or BRCA2 mutation compared to chemotherapy (CT). The objective of this prospective study was to evaluate the clinical benefit of PARPi treatment in terms of response, outcomes and survival by breast cancer type and treatment in a Latin-American population.

**Methods:**

From September 2019 to April 2023, we analyzed the data of patients with HER2-ve ABC with germline and/or somatic mutation of BRCA1 or BRCA2, or in the homologous recombination repair genes, treated with olaparib or talazoparib in daily clinical practice by oncologist from Argentina and México. real-world objective response rate (rwORR), best response rate, real-world progression-free survival (rwPFS) and real-world overall survival (rwOS) were analysed with R software and RStudio version 14.0.

**Results:**

After a median follow-up of 18.07 months (95% CI 10.53–30.07), 51 patients were treated with PARPi. Mean age at starting treatment was 47.08 years. 62.7% had ER + ve/HER2-ve and 35.3% had triple negative breast cancer (TNBC). 62.7% and 37.3% of patients received talazoparib and olaparib, respectively. BRCA 1 and 2 germline mutations were the most common alterations found in 96% of patients. 37.5% of patients received platinum-based CT in the (neo)adjuvant/metastatic setting. At the time to starting PARPi treatment, 57.5% had visceral metastasis, the median number of metastatic sites was 2 (range 1–4), the median number of lines was 2 (range 0–8), and 23.5% and 31.4% received PARPi in the 1st line and 2nd line, respectively.

The rwORR was 47.0%, and the median real-world progression-free survival-1 (rwPFS_1_) was 7.77 months (95% CI 5.67–14.7). There was a tendency of better rwPFS_1_ in patients with versus without previous CT in the advance setting, 6.37 months (95% CI 5.03–8.73) and 14.30 months (95% CI 6.47–NR), respectively (*p* 0.084). The median rwOS was 26.97 months (95% CI 13.50–NR) and higher in the subgroup of patients naïve for CT versus previous exposure to CT in the advance setting, the median rwOS was 32.1 months (95% CI 27.0–NR) versus 13.0 (95% CI10.1–NR), respectively (*p* 0.022). The medium real-world progression-free survival-2 (next treatment after PARPi failure) was 4.00 months (95% CI 3.43–7.13). Treatment was discontinued due to adverse events in 4.0% of patients.

**Conclusion:**

This is the first evidence in a Latin-American population that replicates the data already published in randomised clinical trials and other scanty real-world evidence studies in this field, showing positive results in rwORR and rwPFS, and encouraging data in rwOS. Notably, there was a high proportion of patients with visceral progression even with visceral crisis and need for CT. Interestingly, there were similar rwOS results among subgroups (TNBC versus ER + ve/HER2-ve, talazoparib versus olaparib, etc).

## Introduction

Approximately 5%–10% of breast cancer (BC) patients carry germline mutations in BRCA1 or BRCA2 genes (gBRCA1/2m), tumor suppressor genes that synthetise proteins that are essential for the recognition and subsequent high-fidelity repair of DNA double-strand breaks by homologous recombination repair machinery (HRR) [1].

The detection of homologous recombination deficiency (HRD) tumors lies in the potential benefit from DNA-damage repair (DDR) targeted agents, however, their efficacy outside the context of BRCA1 and BRCA2 mutations is still under investigation. Approximately 35% of triple negative (TN) tumors exhibit HRD, which makes them particularly sensitive to drugs that act through DNA damaging [2].

Over the past few years, many studies have been focused on developing companion molecular tests that detect and quantify HRD, hence, predict tumor response to platinum agents and/or HRD-targeted treatment, but to date, they have not yet been validated [3]. 

Deficiency in BRCA1/2 results in HRD, and this confers a particularly sensitivity to poly(adenosine diphosphate-ribose) polymerase inhibitors (PARPi) in early and advanced disease. Based on EMBRACA and OlympiAD studies, two PARPi: talazoparib and olaparib, respectively, obtained U.S. Food and Drug Administration approval for the treatment of gBRCA1/2m HER2 negative (HER2-ve) advanced breast cancer (ABC) [4, 5].

To date, germline pathogenic or likely-pathogenic mutations of BRCA1/2 genes are the only predictive genetic biomarkers of response to PARPi [6]. Little is known about somatic genetic biomarkers (from the broad family of genes related to DDR) predictors of response or resistance originating in biopsy samples of metastasis or circulating tumor DNA [7]. Limited data are available worldwide on the effectiveness and patient predisposition of PARPi in daily practice. 

The aim of this collaborative prospective real-world evidence study is to evaluate the effectiveness, patient´s profile characterisation, patient predisposition description, treatment sequence of PARPi, and treatment beyond PARPi progression in patients with HER2-ve ABC, who have germline and/or somatic mutation in BRCA1/2 genes, or HRR genes treated with olaparib or talazoparib. The study recruited patients principally from an Argentina and México collaboration.

## Patients and methods

Being a real-life, observational, prospective, non-comparative, non-intervention study, as many patients as possible were included consecutively as long as they met the inclusion criteria.

A confidential list was maintained which allowed the association of the coding assigned to the patient and the identified data. We maintained the rules of the International Conference on Harmonization Good Clinical Practice and the applicable local regulations. The trial protocol was approved by ethics review committees at all participating institutions/countries.

Patients included had to comply with the following *inclusion criteria*: a) to be aged ≥18 years, b) histological and immunohistochemical diagnosis of hormone-receptor positive and HER2-ve BC or hormone-receptor negative (<1%) and HER2-ve BC triple negative breast cancer (TNBC), at primary or metastatic site; c) presence of pathogenic or probably pathogenic germline and/or somatic mutation in BRCA1 and/or BRCA2 genes, or in genes linked to DNA repair by homologous recombination listed here: PALB2, CHEK2, ATM, ATR, NBN, BARDO, BRIP1, RAD50, RAD51C RAD51D, MRE11, PTEN, FANCA, FANCC, FANCD2, FANCE, FANCF, FANCG and FANCL; d) have metastatic or locally advanced unresectable disease; and e) have completed at least one course (28 days) of treatment with olaparib or talazoparib approved by local agencies.

The following were considered as exclusion criteria: a) insufficient data in the clinical chart for prospective evaluation, b) not having completed at least one cycle (28 days) of treatment with a PARPi, c) HER2-receptor positive BC, and d) absence of germline or somatic mutations in predefined genes. Patients were not required to be receiving PARPi therapy for any minimum period of time.

Tumour assessments were conducted in accordance with local practice and standard of care at each patient visit.

### Study design and population

***LuciA-15*** is a collaborative prospective, observational, non-comparative (one cohort) study for patients treated with olaparib or talazoparib with HER2-ve ABC, who carry pathogenic or likely-pathogenic germline and/or somatic mutations in BRCA1 and/or BRCA2 genes, or in genes related to HRR mechanism.

The standard and basic determination to indicate olaparib or talazoparib in daily practice is the presence of pathogenic or likely-pathogenic germline mutation of the BRCA1/2 genes by the Next Generation Sequencing (NGS) technique. The genetic tests for each patient were indicated at the discretion of the treating physician and/or genetic counsellor, and covered out of pocket by the patient, their insurance or access programs offered by the pharmaceutical industry.

Considering this, there were differences in the number of genes, panels and/or germline or somatic alterations found that did not allow further subgroup analysis. The aim of this study was to evaluate the effectiveness and identify patient´s profile to improve the management and understanding of PARPi use in ABC in daily clinical practice.

The *primary objectives* of this study: real-world objective response rate (rwORR), real-world progression-free survival (rwPFS) on PARPi treatment real-world progression-free survival-1 (rwPFS_1_) and real-world overall survival (rwOS). The *secondary objectives* were: rwPFS after PARPi progression real-world progression-free survival-2 (rwPFS_2_), progression-free and survival rates (SRs) at 6, 12, 18, 24 and 30-months, patient predisposition, patterns of progression, and first treatment beyond progression.

Based on the number of patients included, subgroup analysis was considered, the outcomes depended on BC subtype, previous exposition or not to platinum-based CT (PbCT) and general chemotherapy (CT), etc.

### Data source and extraction

Prospective collection of patient´s data loaded in the electronic or paper clinical chart, and reports of imaging studies and data were provided by the treating oncologists. National collaborations were requested to improve recruitment, considering the infrequent indication of PARPi in ABC. Demographic, epidemiological, clinical-pathological and patient predisposition data were collected and detailed in Table 1.

Genetic data collected: name of gene (BRCA1, BRCA2, PALB2, CHEK2, ATM, ATR, NBN, BARD, BRIP1, RAD50, RAD51C, RAD51D, MRE11, PTEN, FANCA, FANCC, FANCD2, FANCE, FANCF, FANCG, FANCL, others), type of mutation (pathogenic or likely-pathogenic, others), and genetic tests requested (germline and/or somatic, individual genes, panels or platforms like FoundationOneCDx).

Data collected for the effectiveness analysis were rwORR, the best responses achieved in the first 6 months of treatment based on RECIST version 1.1 (https://recist.eortc.org/recist-1-1-2/) adapted by each oncologist decision; rwPFS_1_, rwPFS_2_, rwOS, progression-free rate (PFR) and SR at 6, 12, 18, 24 and 30 months. No data regarding side effects were collected. Definitions of all clinical outcome variables are presented in Table 2.

A periodic review of health records by physicians was carried out to ensure data quality control. The study was closed (March, 2023) at the discretion of the research team, after a plateau of no new patients included.

### Ethical considerations and data safety

The present study has no impact on patient safety from a clinical point of view. The data obtained in this study was strictly confidential, no initial or subsequent contact with patients was performed. The study was developed under strict supervision of the ethical principles outlined in the Declaration of Helsinki, rules of Good Clinical Practices and applicable legislation. The final protocol of the study was approved by the Ethics Committee of the Instituto Alexander Fleming, Buenos Aires, Argentina.

### Statistical analysis

The present study included descriptive analyses, in which means, medians, SDs, percentiles and ranges were used. Categorical variables were described by proportions. Where appropriate, the 95% confidence interval was presented at both ends. The data will be censored at the last follow-up if the patient is alive. Logistic regression and Kaplan-Meier models were used for survival analyses. Statistical analyses will be performed with R software and RStudio version 14.0.

## Results

Between September 2019 and April 2023, 51 patients were included for analysis (50 patients from Argentina and 1 patient from Mexico). At the time to start PARPi the median number of metastatic sites was 2 (range 1–4), most of the patients had exclusive visceral disease (*n*: 27, 57.0%), followed by bone only disease (*n*: 7, 14.0%) and ≥2 metastatic sites (*n*: 8, 17.0%). The ECOG-PS was 0 and 1 in 60.8% (*n*: 31) and 35.3% (*n*: 18), respectively. All the detailed data is summarised in Table 2.

Regarding genetic tests performed, based on oncologist personal decision and/or genetic counseling, from 50 patients´ data, 96% of alterations found were germline mutation (one patient with somatic mutation and one patient with concurrent germline and somatic pathogenic mutation), and gBRCA2 follow by gBRCA1 mutations were the most common mutations encountered, 52% and 42%, respectively. One patient tested by somatic comprehensive gene panel evidenced concomitant pathogenic alteration in sATM, sRAD51c and sBIR-I genes. The most common genetic assays performed were 2-genes by NGS with or without MLPA (germline BRCA1/2) follow by 26, 30 or 33-genes panel, and three patients were tested by FoundationOneCDx.

### Treatment patterns

At the time of final analysis (March 2023), PARPi treatment was ongoing in 16 patients (31.4%)**.** Talazoparib was the first PARPi approved for commercialisation in Argentina in 2019, followed by Olaparib in 2020. Talazoparib was indicated in 62.7% (*n*: 32) and olaparib in 37.3% (*n*: 19) of patients, only one patient with a TNBC treated with olaparib in second line was rechallenged in 6th line with talazoparib.

The median number of lines for advanced setting before PARPi indication was 2 (range 0–8). In the general population the most frequent indication of PARPi was in early lines of ABC, first line 23.5% (*n*: 12), second line 31.4% (*n*: 16), third line 29.4% (*n*: 15), and four lines and beyond 15.7% (*n*: 8). Similar rate of use of PARPi before or after CT for ABC, 53.2% and 46.8%, respectively.

The most common starting dose was the recommended daily full dose for talazoparib 1 mg and olaparib 600 mg (*n* = 48; 94.1%), only three patients (5.9%) started with the first reduction dose. At least one dose adjustment was seen in 14 patients (29.2%), most frequently reduction from 1 to 0.75 mg for talazoparib (*n* = 9) and 600–450 mg for olaparib (*n*:4). At least one dose interruption was seen in 16 patients (33.3%), most frequently with talazoparib (*n*: 14) than olaparib (*n*: 2). Treatment was discontinued due adverse events in 2 patients (4.0 %).

### First treatment beyond PARPi progression

In the first 6 months of treatment, 23.5% (*n*: 12) of patients progressed under talazoparib or olaparib treatment, and at the time of final analysis 76.5% (*n*: 39) of patients progressed. Visceral disease (*n*: 11, 32.4%) and ≥2 metastatic sites (*n*:9, 26.5%) were the most frequent sites of progression leading to CT (mono or in combination) as the treatment of choice (*n*:21, 55.3%), and one out of four with platinum-based schemes (*n*: 5). Furthermore, visceral crisis by oncologist criteria as progression event was common, data available from 36 patients showed 27.8% (*n*: 10) of cases.

### Outcomes

On March 2023, after a median follow-up of (IQR): 18.0 months (95% CI 10.5–30.0), 51 patients were treated with PARPi, 23.5% of them still on treatment with PARPi (*n*: 12), and 76.5% of patients progressed (*n*: 39), 39.2% (*n*: 20) had died, and 9.8% were lost on follow-up (*n*: 5). The rwORR, calculated based on the physician’s assessments as recorded in patient charts, were achieved in 47.0% of patients (*n*: 24), including complete response (CR) in 7.8% (*n*: 4) and partial response (PR) in 39.2% (*n*: 20). The CBR_≥24w_ was 76.4% (*n*: 39) including those patients with SD_≥24w_ of 29.4% (*n*: 15), and one out of four of patients progressed on PARPi during the first 6 months of treatment (*n*:12, 23.5%). One patient with a TNBC who carries the somatic concurrent mutation in ATM, RAD51c and MYH by FoundationOneCDx, progressed at 5.0 months with olaparib treatment in fifth line. Another patient with ER+ve/HER2-ve gPALB2m progressed at 5.7 months with talazoparib in second line. And finally, one patient with ER + ve/HER2-ve sBRCA2m was treated with talazoparib in the first line and progressed at 6.5 months.

**Figure 1.**
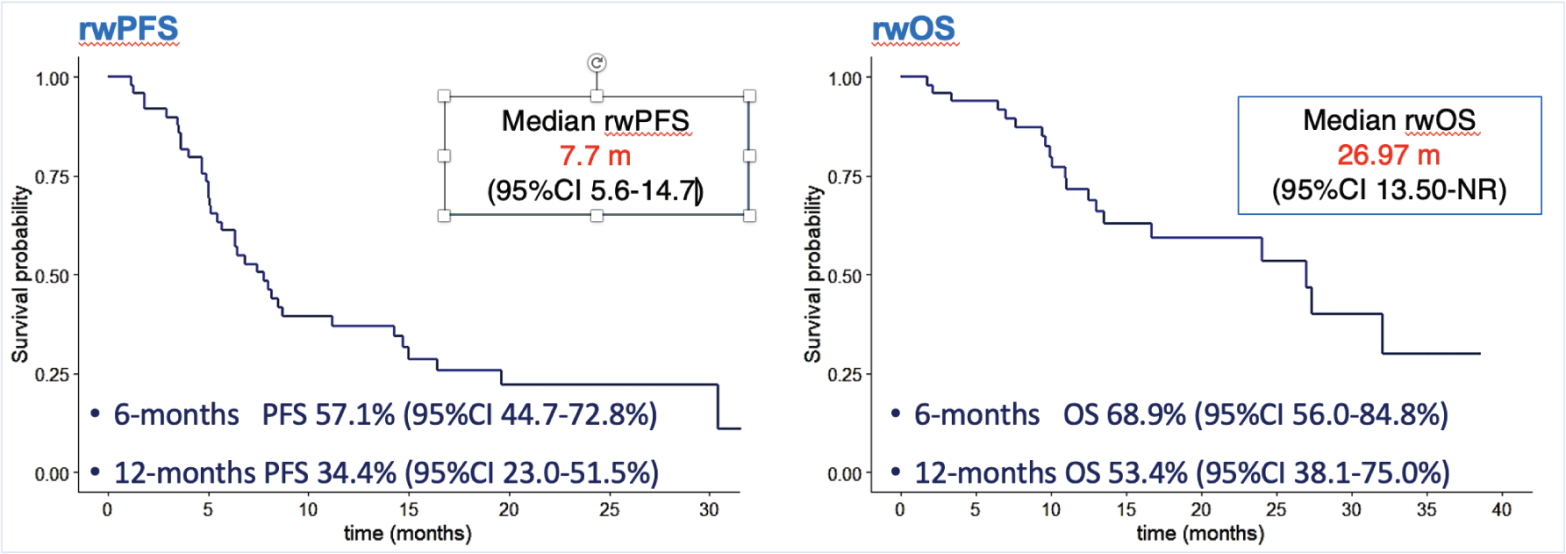
Kaplan–Meier curves in general population. rwPFS, Real-world progression-free survival and rwOS, real-world overall survival. 95% CI, 95% Confidence interval.

**Figure 2.**
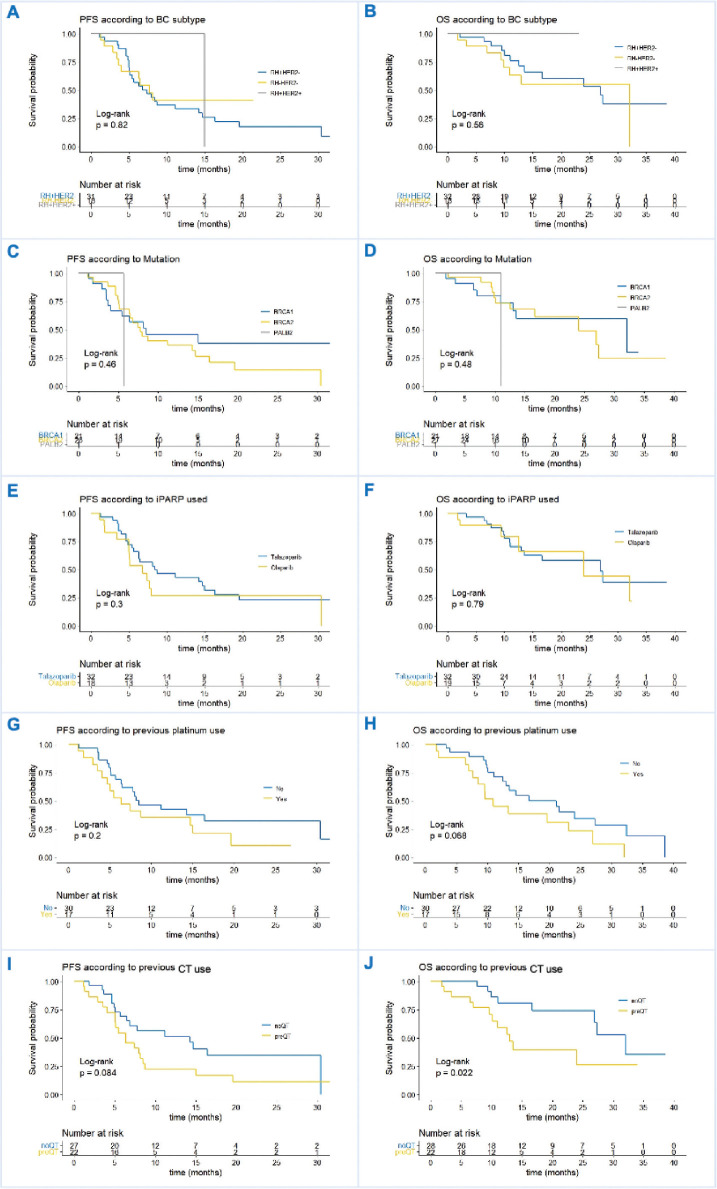
(a and b): rwPFS and rwOS in BC subtype ER + ve/HER2-ve versus TN. (c and d): rwPFS and rwOS in BC based on gene mutation: BRCA1 versus BRCA2 versus PALB2. (e and f): rwPFS and rwOS based on PARPi subtype: talazoparib versus olaparib. (g and h): rwPFS and rwOS based on previous PbCT use yes or no. (i and j): rwPFS and rwOS based on previous CT use yes or no.

Based on real-world evidence studies definitions we performed ways of progression and survival analysis. Two of the primary objectives of the study were analysed the rwPFS_1_ and rwOS in the general population. Due to the limited number of patients included, subgroup analysis was insufficient for conclusions, despite that, we present Kaplan-Meier curves comparing outcomes between BC subtypes. The median rwPFS_1_ was 7.7 months (95% CI 5.6–14.7) and the rwPFS at 6 and 12 months were 57.1% (95% CI 44.7%–72.8%) and 34.4% (95% CI 23.0%–51.5%), respectively. Regarding rwOS in the whole population, the median rwOS was 26.9 months (95% CI 13.5–NR) and the rwOS at 6 and 12 months were 68.9% (95% CI 56.0%–84.8%) and 53.4% (95% CI 38.1%–75.0%), respectively (Figure 1). The statistical analysis showed no differences between BC subtypes, menopausal status, type of gene mutations, previous exposure to PbCT, and type of PARPi utilised. There was a tendency of better rwPFS in patients without versus previous CT in the advance setting, the rwPFS was 14.3 months (95% CI 6.4–NR) versus 6.3 months (95% CI 5.0–8.7), respectively with a *p* value of 0.084. The positive result was found in the subgroup of patients naïve versus previous exposure to CT in the advance setting, the median rwOS was 32.1 months (95% CI 27.0–NR) versus 13.0 (95% CI 10.1–NR), respectively, with a *p* value of 0.022. For other subgroup analysis, there were no differences in rwOS as in rwPFS. (Figure 2).

Analysing in isolation the effect of the use or not of PbCT prior to the start of PARPi had a negative trend in rwOS. When adjusted this effect in a bivariable analysis including the effect of the general CT use for advance setting before PARPi treatment, showed its value is lost. (PbCT HR 1.5, 95% CI 0.6–3.8, *p* 0.36 and overall CT HR 2.5, 95% CI 0.9–6.5, *p* 0.05).

**Figure 3.**
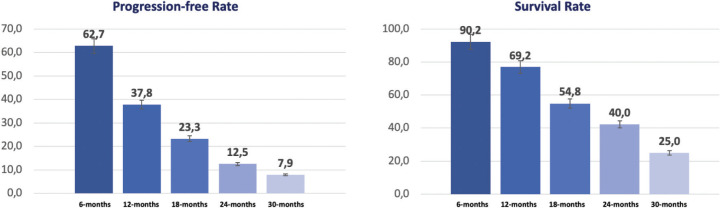
Progression-free and SR at 6, 12, 18, 24 and 30 months.

One of the secondary objectives of the study was evaluating the proportion of patients alive without progression at 6, 12, 18, 24 and 30 months, the progression-free rate; and for the same periods of time the proportion of patients alive, the SR (Figure 3).

Regarding PFR, coinciding with the median PFS extracted in the Kaplan-Meier curves (57.1% at 6 months), 62.7% of patients were alive without evidence of disease progression, descending to 23.3% at 18 months of follow-up. Remarkably, close to 70% of patients were alive at 1-year, and one out of four (25.0%) was alive at 30-months of follow up.

**Figure 4.**
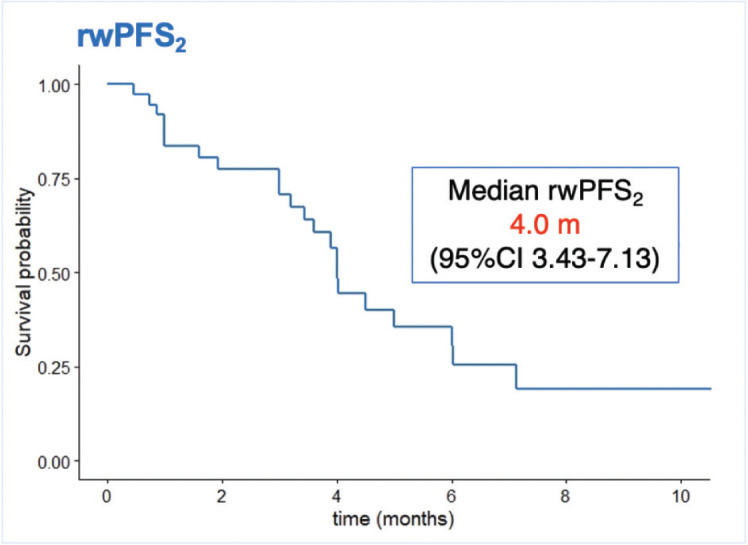
rwPFS_2_, rwPFS with de first or next treatment after PARPi failure.

Another secondary objective analysis was to evaluate the rwPFS with the first or next treatment after PARPi failure, defined as rwPFS_2_. Based on disease distribution, systemic treatment resistance criteria, previous exposure to CT and PbCT, the scenario beyond PARPi progression in our population had a poor prognosis. Notably, 23.5% of patients progressed on PARPi during the first 6 months of treatment, 32.4% with visceral compromise, 27.8% with visceral crisis, and 26.5% with ≥2 metastatic sites. CT alone or in combination was the treatment of choice in most patients (55.3%), and one out of four included CT with platinum-based schemes. Considering all of this, the medium rwPFS_2_ was 4.0 months (95% CI 3.4–7.1) (Figure 4).

## Discussion

***LuciA-15*** trial is the first prospective trial to evaluate the clinical effectiveness of PARPi in patients with BRCA and non-BRCA mutations, HER2-ve ABC in a setting that closely reflects real-life clinical practice. Only three patients were included with non-gBRCAm to reach any conclusion about effectiveness in this subgroup. Not many trials in this field were published, only three real-world studies, two phase II trials, and two major phase III randomised clinical trials (RCTs) that support our current treatment decisions, summarised in Table 3. The ***LUCY*** trial is a pragmatic, open-label, single-arm trial of olaparib, in HER2-ve BRCAm ABC. At the interim analysis, 54.4% received olaparib in 1 L, the median investigator assessed PFS was 8.1 months (95% CI 6.9–8.6), the clinical response rate was 48.6%, the median duration of clinical response was 6.6 months (IQ range, 4.2–10.8), and median time to first subsequent treatment or death was 9.6 months (95% CI 8.6–11.1) [8].

Dawood [9] published one of the few studies available in real-world evidence based on retrospective data extracted from US TriNetX Research Platform, 586 patients with HER2-ve ABC treated with PARPi. Median OS was 23 months, 18.0 months in TNBC and 22.0 months in ER + ve/HER2-ve (*p* 0.37) [9]. Another interesting study was published by Batalini *et al* [10] data were extracted from Flatiron Health – Foundation Medicine Clinico-Genomic Database, assessed outcomes for patients with gBRCAm compared to patients with either sBRCAm or other HRD treated with PARPi. Neither median rwPFS (7.0 versus 5.5 months) nor rwOS (15.0 versus 11.5 months) were significantly different between the non-gBRCA and gBRCA cohorts. For 9 patients with sBRCAm, median rwPFS was 7.1 mos (range 1.4–12.4) and all patients had progressed by data cut off [10]. These findings are consistent with the results from ***TBCRC -048***, a phase II single-arm, two-stage design trial that explored the effectivity of olaparib in ABC with pathogenic mutation in HRR genes, excluding gBRCA1/2m tumors, platinum-refractory disease or >2 L of CT for ABC. In the germline mutated cohort (*n*: 27) the primary endpoint ORR was 33% (all PR) and the CBR_18w_ was 67%. Notably, all PR were reached in gPALB2 (ORR, 82%) with CBR_18w_ of 100% and a median PFS of 13.3 months (90% CI 12–NA). In the somatic mutated cohort (*n*: 26) the ORR was 31% and the CBR_18w_ of 44%. All PR were achieved in sBRCA1/2m carriers (ORR, 50%) and the median PFS of 6.3 months (90% CI 4.4–NA). No responses were observed with ATM or CHEK2 mutations alone [11]. The talazoparib beyond BRCA (TBB) is an open-label phase II trial, to evaluate talazoparib in patients with pretreated HER2-ve ABC (*n* = 13) or other solid tumors (*n* = 7) with mutations in HRR genes other than *BRCA1* and *BRCA2*. In patients with BC the ORR was 31% and the CBR_≥6m_ was 54%. All patients with gPALB2m had treatment-associated tumor regression [12].

In the randomised phase III ***OlympiAD*** trial, olaparib monotherapy significantly increased PFS compared with CT treatment of physician’s choice in patients with gBRCAm, HER2-ve ABC. The median PFS was 7.4 months versus 4.2 months with a HR 0.58 (*p* < 0.001). The phase III ***EMBRACA*** trial compared talazoparib to the physician’s choice of CT in gBRCAm patients with unresectable or ABC. The median PFS was 8.6 months for those treated with talazoparib compared with 5.6 months in those treated with CT (HR 0.54, *p* < 0.0001) with an ORR of 62.6% with talazoparib compared with 27.2% with CT [14]. 

LuciA-15 is the first prospective real-world evidence in a specific subgroup of patient that treatment decision-making is based on specific biomarkers. This is the first evidence in Latin-American population, with appropriate follow-up and good quality and complete data collected. A limited number of patients included in our trial and few number of patients with non-BRCAm tumors did not allow subgroup analysis with statistical value.

## Conclusion

In conclusion, LuciA-15 adds important and valuable evidence on real life PARPi management and patient profile in ABC, and reinforces the hard data already published in RCTs. More evidence is necessary to expand the indication in germline or somatic non-BRCAm tumors, in the meantime case by case discussion with a multidisciplinary team and molecular tumor board is mandatory.

## Conflicts of interest

The authors declare that they have no conflict of interest.

## Funding

This work received no specific grant from any funding agency.

## Figures and Tables

**Table 1.** Endpoint definition.

Real-world objective response rate	Definition/variables
CR	No clinical or by images evidence of disease after start PARPi treatment (at any time, no 24-week minimum)
PR	Partial reduction in tumor burden in some or all areas without any areas of increasing disease. rwPR captures a decrease in disease burden though disease is still present
Stable disease ≥24 weeks (SD_≥24 w_)	Patients remained on PARPi for a minimum of 24 weeks without complete or PR, death, treatment switch, or progression
Stable disease <24 weeks (SD_<24w_)	Stable disease recorded for initial response, with a subsequent progression recorded after <24 weeks or treatment switch for reason other than progression after <24 weeks, or death without recorded progression after <24 weeks
rwORR	Proportion of patients achieving a complete or PR as assessed by the physician and reported in the patient records
Clinical benefit rate ≥24 weeks (CBR_≥24w_)	Proportion of patients who achieved a complete, PR and had stable disease for ≥24 weeks as assessed by the physician
Progressive disease <24 weeks (PD_<24w_)	PD recorded from start of PARPi treatment to <24 weeks. Progression could be radiological (e.g., RECIST), symptomatic, or clear progression of non-measurable disease, as long as progression could be documented from start of PARPi treatment to <24 weeks.
Progressive disease (PD)	PD recorded from start of PARPi treatment to last visit, death or lost of follow-up.
Clinical outcomes	
rwPFS_1_	Time from start of PARPi treatment to date of progression, death for any cause, or date of start next treatment or last visit reported in the medical chart if the patient had not progressed or died.
rwPFS_2_	Time from start next treatment after PARPi to death for any cause, clinical progression, loss of follow-up o date of start next treatment, only for those patients with disease progression on PARPi
rwOS	Time from start of PARPi treatment to death for any cause or loss of follow-up or alive at last visit.
PFR	Proportion of patients with no evidence of progression or death at 6, 12, 18, and 24 months
SR	Proportion of patients alive at 6, 12, 18, and 24 months

**Table 2.** Baseline characteristics.

Patient characteristics	*n*: 51 *p*	%
Female Male	50 1	98.0 2.0
Mean age (years)	47.08	23–83
Premenopausal	26	52.0
Meta or synchronic bilateral BC^a^	4	8.0
BC subtype	*n*: 51 *p*	%
ER+/HER2- TN ER+/HER2+	32 18 1	62.7 35.3 2.0
Disease-free interval^b^	*n*: 47 *p*	%
De novo stage IV or ≤12-months Relapse >12-months	17 30	36.2 63.8
sPIK3-CA status^c^	*n*: 26 *p*	%
Wild-type Mutated	19 7	73.1 26.9
Previous treatment	*n*: 51 *p*	%
Neoadjuvant and/or adjuvant CT Anthracyclines Taxanes Anthracyclines + taxanes CT + platinum-based CT + capecitabine Others	30 3 2 15 4 2 4	75.0 10.0 6.7 50.0 13.3 6.7 13.3
Adjuvant endocrine therapy	*n*: 19 *p*	%
Tamoxifen Aromatase inhibitors	14 5	73.7 26.3
Advanced CT lines	*n*: 50 *p*	%
0 lines 1 line 2 lines 3 lines Median lines of ABC CT	28 16 4 2 0	56.0 32.0 8.0 4.0 0–3
Advanced endocrine therapy lines	*n*: 35 *p*	%
0 lines 1 line 2 lines 3 lines Median Lines of ABC ET	6 17 11 1 1	17.1 48.6 31.4 2.9 0–3
ET + CDK4/6 inhibitors	28	82.4
ET + mTOR inhibitors	7	20.6
Alpelisib	2	6.1
Fulvestrant	15	44.1
ICP inhibitors	1	5.0
Number of lines before PARP inhibitors		
0 lines 1 line 2 lines 3 lines ≥4 lines Median lines before PARPi	12 16 15 2 6 2	23.5 31.4 29.4 3.9 11.8 0–8
PARP inhibitor indicated		
Talazoparib Olaparib	32 19	62.7 37.3
PbCT in (neo)adjuvant or advance setting	18	37.5
PbCT in the advance setting pre-PARPi	13	25.5
PARP inhibitors before or after CT in ABC		
PARP inhibitors before CT PARP inhibitors after CT	25 22	53.2 46.8
Platinum-free interval		
≤6 months >6 to 12 months >12 months	9 5 4	50.0 27.8 22.2
Metastatic sites		
Bone Visceral More than two Soft tissue CNS w/wo others	7 27 8 5 1	14.9 57.4 17.0 10.6 6.7
Number of metastatic sites		
1 2 3 4 Median number of metastatic sites	17 19 14 1 2	33.3 37.3 27.5 2.0 1–4
ECOG performance status		
0 1 ≥2	31 18 2	60.8 35.3 4.0
Concomitant RT with PARP inhibitors	9	17.6

a 3 *p* with billateral BC with different phenotypes

b 6 *p* unknown

c 3 *p* unknown

ICP, immune-check points inhibitors. CT, chemotherapy. ET, endocrine therapy. ABC, advanced breast cancer. RT, radiation therapy

**Table 3.** Available studies in ABC with PARPi.

Study	n	PARPi	Subgroups	Line	ORR (%)	PFS (Median in months, 95%IC)	OS (Median in months, 95% IC)
LUCY [8]	252	Olaparib	g/sBRCA1/2m	54% 1 L	48.6	8.1 (6.9–8.6)	NA
Dawood [9]	586	Olaparib talazoparib others	g/sBRCA1/2m	31% 1 L 51% 2 L 30% ≥3 L	NA	NA	23.0 (NA)
Batalini *et al* [10]	62	NA	No gBRCAm gBRCAm	NA	NA	7.7 (NA) 5.5 (NA)	15.0 (NA) 11.5 (NA)
TBCRC-048 [11]	20	Olaparib	g/sBRCA1/2m g/sHRRm	≥3 L	33.0	13.3 (12–NA)	NA
TBB [12]	13	Talazoparib	g/sHRRm	NA	31.0	NA	NA
OlympiAD [13]	302	Olaparib	gBRCA1/2m	29% 1 L	59.9	7.4 (NA)	19.3 (NA)
EMBRACA [14]	431	Talazoparib	gBRCA1/2m	39% 1 L 37% 2 L 24% ≥3 L	62.6	8.6 (7.2–9.3)	19.3 (16.6–22.5)
LuciA-15	51	Olaparib talazoparib	g/sBRCA1/2m g/sHRRm	23.5% 1 L 31.4% 2 L 45.1% ≥3 L	47.0	7.7 (5.6–14.7)	26.67 (13.5–NR)

PARPi, PARP inhibitor; g/sBRCA1/2, germline/somatic BRCA 1 or 2 mutations; g/sHRR, germline/somatic Homologous Recombination Repair genes mutation; ORR, objective response rate; PFS, pprogression-free survival; NA, not available or not specified; NR, not reached
